# Main control factors affecting mechanical oil recovery efficiency in complex blocks identified using the improved k-means algorithm

**DOI:** 10.1371/journal.pone.0248840

**Published:** 2021-05-04

**Authors:** Qiuyu Lu, Suling Wang, Minzheng Jiang, Yanchun Li, Kangxing Dong

**Affiliations:** School of Mechanics Science & Engineering, Northeast Petroleum University, Daqing, Heilongjiang, China; University of Defence in Belgrade, SERBIA

## Abstract

The system efficiency of pumping units in the middle and late stages of oil recovery is characterized by several factors, complex data and poor regulation. Further, the main control factors that affect system efficiency in different blocks vary greatly; therefore, it is necessary to obtain the block characteristics to effectively improve system efficiency. The k-means algorithm is simple and efficient, but it assumes that all factors have the same amount of influence on the output value. This cannot reflect the obvious difference in the influence of several factors in the block on the efficiency. Moreover, the algorithm is sensitive to the selection of the initial cluster centre point, so each calculation result that reflects the efficiency characteristics of the block system cannot be unified. To solve the aforementioned problems affecting the k-means algorithm, the correlation coefficient of all the factors was first calculated, followed by extracting the system efficiency of the positive and negative indicators of standardization. Next, the moisture value was calculated to obtain the weight of each factor used as a coefficient to calculate the Euclidean distance. Finally, the initial centre point selection of the k-means algorithm problem was solved by combining the dbscan and weighted k-means algorithm. Taking an oil production block in the Daqing Oilfield as the research object, the k-means and improved algorithm are used to analyse the main control factors influencing mechanical production efficiency. The clustering results of the two algorithms have the characteristics of overlapping blocks, but the improved algorithm’s clustering findings are as follows: this block features motor utilization, pump efficiency and daily fluid production, which are positively correlated with system efficiency. Further, low-efficiency wells are characterized by the fact that the pump diameter, power consumption, water content, daily fluid production, oil pressure and casing pressure are significantly lower than the block average; high-efficiency wells are characterized by pump depths lower than the block average. For this block, it is possible to reduce the depth of the lower pump and increase the water-injection effect to increase the output under conditions of meeting the submergence degree, which can effectively improve the system efficiency.

## Introduction

In most areas of China, the average efficiency of pumping unit wells is 12–23%. In the United States, the average efficiency of pumping unit wells is relatively higher, but it does not exceed 45% [1]. It is clear that there is still significant room for improvement in pumping unit system efficiency, especially in China. The primary reason for the low efficiency of pumping well systems is that the load changes greatly during the energy-transfer process from the motor to the pump during operation, which induces a large amount of loss [2, 3]. Further, many factors influence the efficiency of pumping unit systems in the middle and late stages of oil production; the data surrounding this is complex and uncharacterized, resulting in poor system efficiency control. With the rapid development of digital oilfields, a large amount of monitoring data has become available regarding mechanical production management systems [4]. Data mining technology can extract unknown hidden correlations that have potential application value from a large amount of noisy practical data, and convert this data into useful information [5, 6]. At present, AI technology has developed into various fields [7, 8], data mining technology has gradually matured, and the application frequency of rough set theory, neural network, and cluster analysis is extremely high [9–11]. At present, many scholars have applied data mining technology to the oilfield industry, mainly in many aspects such as water injection optimization, production forecasting, and enhanced oil recovery in the oil industry [12–15]. Among many data mining techniques, cluster analysis method as an effective data analysis method has achieved good application in reservoir description and downhole condition diagnosis of oilfield development [16]. However, cluster analysis is less applied in the efficiency of block pumping unit system. The K-means clustering algorithm is more suitable for the efficiency analysis of the oil pumping unit system due to its simplicity and linear time complexity [17, 18]. Therefore, it is of great significance to apply the clustering algorithm effectively to improve the efficiency of the block pumping unit system.

The k-means algorithm is sensitive to the selection of the initial cluster centre point, and uses Euclidean distance to measure the similarity between clusters, which does not reflect the characteristics of the data itself. At present, many scholars have proposed improvements to the k-means algorithm. The Rk-means algorithm, proposed by Lei [19], uses an improved MaxMin initialization method to overcome the sensitivity to the initial cluster centre, and can automatically segment and merge clusters. Reda [20] combined the random forest and wk-means algorithm to build a hybrid framework that can overcome the shortcomings of misuse and anomaly detection. Manoharan [21] proposed an optimized k-means centre of gravity initialization method; the algorithm uses the divide-and-conquer method to find the initial centre and attribute the data to the appropriate cluster. The improvement of the existing k-means algorithm is mostly to overcome the sensitivity of clustering centers. For complex oilfield data and multiple factors, the existing algorithms still have certain limitations.

The contribution of this paper is to improve k-means based on the characteristics of oilfield data. When determining the optimal number of clusters, this study considers the differences between clusters and the similarities within clusters of the k-means algorithm, and eliminates the effect of the number of clusters and sample size on the calculation results. It is proposed that the weighted Euclidean distance be used to calculate the distance between the data sample and the cluster centre, which can better combine the characteristics of the data itself. Because most of the cluster centres are distributed in the range of high data object density, the Dbscan algorithm is used to extract the initial cluster centres. Using the weighted k-means algorithm combined with density clustering, the block oilfield data is clustered and analysed, and the block pumping well characteristics are extracted, providing an effective basis for subsequent analysis, solve the sensitive problem of cluster center. This paper uses an improved algorithm to analyze the ground part and downhole part of the block oilfield data, then compares the application effects of the k-means algorithm and the improved algorithm in the block oilfield data, and makes a visual comparison, it proves that the improved algorithm is more suitable for cluster analysis of block oil fields. Based on the improved algorithm to excavate the efficiency characteristics of the block pumping unit system, and analyze from three aspects: motor parameters, downhole parameters and operating parameters, combine analysis to find out the obvious characteristics of low-efficiency wells and high-efficiency wells, and summarized measures to improve the efficiency of the block system. This result is of great significance to the research on improving the efficiency of the block pumping unit system.

## Parameter selection and data collection on the efficiency of pumping units

### Parameter selection

The formula for calculating the power of the pumping unit is as follows:

$$\eta =\frac{Q\left[f_{w}\rho_{w}+\left(1-f_{w}\right)\rho_{o}\right]\left[H_{a}+\frac{\left(F_{a}-F_{b}\right)\times 10^{6}}{\left[f_{w}\rho_{w}+\left(1-f_{w}\right)\rho_{o}\right]g}\right]g}{86400P_{1}}\times 100\%$$ (1)

where *η* is the efficiency of the pumping well system; *P*_*1*_ is the motor input power; *Q* is the daily production of the oil well; *H*_*a*_ is the liquid depth; g is the gravitational acceleration; *F*_*a*_ is the oil pressure; *F*_*b*_ is the casing pressure; *f*_*w*_ is the moisture content; *ρ*_*w*_ is the water density; and ρ_o_ is the oil density.

The aforementioned equation can directly obtain the efficiency of the pumping unit. It can be seen that the factors that directly influence the efficiency of the pumping unit system are the daily output of the oil well, the input power of the motor, the density of the pumped liquid, the water content, the depth of the dynamic liquid surface and the oil pressure at the wellhead. However, the indirect factors affecting the efficiency of beam pumping units are not addressed. Some scholars have studied the factors that influence the efficiency of the pumping unit system from both the surface and the downhole [22, 23] perspectives–the factors that affect pump efficiency include pump depth, pump diameter, sinking degree, daily liquid production and crude oil density; the factors that affect the power of the polished rod of the pumping unit include rated power, active power and current. However, the factors on the surface and downhole portions influence each other, so several factors must be considered comprehensively. For example, the depth of the dynamic liquid level is equal to the difference between the pump depth and the sinking degree; therefore, the latter two are indirect factors that affect the efficiency of the system. Further, the degree of balance will affect the system efficiency of the pumping unit to a certain extent–the degree of balance is determined by the maximum current of the upstroke and the maximum current of the downstroke. Therefore, the up- and down-stroke current are also indirect factors affecting the efficiency of the pumping unit systems. This study combines motor data and downhole data, dividing the factors that affect the efficiency of the pumping unit system into three categories: motor parameters, operating parameters and downhole parameters. The specific parameters are listed in Table 1.

**Table 1:** Selection parameters for block system efficiency research.

Motor parameters			Operating parameters			Downhole parameters		
Characteristic attributes	Letter code	Unit	Characteristic attributes	Letter code	Unit	Characteristic attributes	Letter code	Unit
**Upstroke maximum current**	**I**_**u**_	**A**	**Stroke**	**s**	**m**	**Liquid depth**	**H**_**a**_	**m**
**Downstroke maximum current**	**I**_**d**_	**A**	**Frequency**	**n**	**min**^**-1**^	**Pump setting depth**	**H**_**b**_	**m**
**Motor input power**	**P**_**1**_	**KW**	**Moisture content**	**f**_**w**_	**%**	**Submergence**	**L**	**m**
**Power consumption**	**p**_**e**_	**KW**	**Daily fluid production**	**Q**	**m**^**3**^**/d**	**Pump diameter**	**Φ**_**b**_	**mm**
**Motor utilization**	**η**_**d**_	**%**	**Casing pressure**	**F**_**b**_	**MPa**	**Pump efficiency**	**η**_**b**_	**%**
			**Oil pressure**	**F**_**a**_	**MPa**			

### Data collection

The data collected here is the production data of a certain block of an oil production plant in Daqing (see Table 2 for details). It can be seen that the values of the original data are quite different, and they include direct and indirect factors that influence system efficiency. This data must be standardized before performing cluster analysis to obtain more accurate and objective results.

**Table 2:** Statistical value of 16 indicators in the block.

	Min	Max	Mean	SD
**F**_**a**_	**0.1**	**0.4**	**0.4**	**0.1**
**F**_**b**_	**0**	**0.4**	**0.4**	**0.1**
**Q**	**2.8**	**39.4**	**42**	**19.6**
**f**_**w**_	**80.4**	**95.5**	**95.1**	**2.3**
**L**	**5.8**	**238.1**	**239.5**	**85**
**s**	**2**	**3**	**3.1**	**0.5**
**n**	**2**	**6**	**5.9**	**1.3**
**Φ**_**b**_	**38**	**70**	**63.7**	**9.5**
**H**_**b**_	**633.7**	**955.1**	**945.9**	**60.6**
**H**_**a**_	**153.3**	**706.6**	**706.4**	**79.9**
**I**_**u**_	**11**	**41**	**43.7**	**15.2**
**I**_**d**_	**10**	**38**	**40.6**	**13.3**
**P**_**1**_	**18.5**	**37**	**36.5**	**9.6**
**η**_**b**_	**6.2**	**47.5**	**48.8**	**16.2**
**η**_**d**_	**6.8**	**26.8**	**28.6**	**11.1**
**P**_**e**_	**2.3**	**9.3**	**9.9**	**3.5**

## Establishing a weighted k-means model combined with Dbscan

### K-means algorithm

The k-means algorithm is one of the ten classic data mining algorithms, and is a distance-based clustering algorithm. It is simple, efficient and does not require range constraints on the data. It can obtain more accurate clustering results for mutually independent data. The flow of the k-means algorithm is as follows:

Step 1: Input the data set *X*, the number of clusters *K*, and randomly select *k* data objects from the data set *X* as the initial cluster centres;

Step 2: Using formula (2), calculate the distance from each sample *x*_*m*_ in the dataset to the cluster centre point *c*_*i*_;

$$dis\left(x_{m},c_{i}\right)=\sqrt{{\left(x_{m}-c_{i}\right)}^{2}}$$ (2)

Step 3: Find the minimum distance from each object *x*_*m*_ to the cluster centre *c*_*i*_, and classify *x*_*m*_ into the same class as *c*_*i*_;

Step 4: Use formula (3) to recalculate and update the cluster centre of each cluster, where *N* is the number of samples in the *k-th* cluster;

$$c_{i}^{'}=\frac{\sum_{i=1}^{N}x_{i}}{N}$$ (3)

Step 5: Repeat steps 2–4 until all cluster centres no longer change or the maximum number of runs is reached.

### Weighted k-means algorithm combined with density clustering

While the k-means algorithm has the advantages of simplicity and efficiency, it also has disadvantages such as difficulty in selecting the K value, an inability to reflect the characteristics of the data and the randomized selection of the initial clustering centre, resulting in different clustering results. This article has made some improvements to the k-means algorithm to mitigate the abovementioned shortcomings, as follows.

#### Selection of the number of clusters

When determining the number of clusters, to minimize the sum of squared errors between groups, it is necessary to make the differences between groups as large as possible. The sum of squared errors within the group (*λ*_*sse-wc*_) reflects the similarity within the group; the sum of squared errors between groups (*λ*_*sse-bc*_) reflects the differences between different groups. To eliminate the influence of the number of clusters and sample size on the calculation results, the formula for determining a reasonable number of clusters can be written as follows:

$$q=\frac{\lambda_{sse-bc}}{K-1}/\frac{\lambda_{sse-we}}{n-K}$$ (4)

where *q* is the coefficient for determining the number of clusters; *λ*_*sse-bc*_ is the sum of squared errors between groups; *λ*_*sse-wc*_ is the sum of squares of errors within the group; *K* is the number of clusters; and *n* is the sample size.

#### Weight calculation

The k-means algorithm assumes that all factors have the same influence on the output value. However, practically, there are obvious differences in the effects of many factors on efficiency, so an additional coefficient is used in the calculation of the Euclidean distance in the improved algorithm.

First, the different factors are standardized through the homogenization of heterogeneous indicators, that is, the influence of many factors on the output value is divided into positive and negative indicators. Second, the entropy of the standardized values is calculated. The larger the entropy value, the higher the disorder of the information, that is, the smaller the utility of the information. The information utility of each indicator depends on the difference between the entropy value of the indicator and 1; the weight of each factor in the comprehensive evaluation is the proportion of its information utility value to the total utility value of all factors. The specific formula for this is as follows:

$$X_{ij}^{\prime }=\left[\frac{X_{ij}-\min\left(x_{1j},\ldots ,x_{nj}\right)}{\max\left(x_{1j},\ldots ,x_{nj}\right)-\min\left(x_{1j},\ldots ,x_{nj}\right)}\right]$$ (5)

$$X_{ij}^{"}=\left[\frac{\max\left(x_{1j},\ldots ,x_{nj}\right)-X_{ij}}{\max\left(x_{1j},\ldots ,x_{nj}\right)-\min\left(x_{1j},\ldots ,x_{nj}\right)}\right]$$ (6)

$$P_{ij}=\frac{X_{ij}}{\sum_{i=1}^{n}Xij}$$ (7)

$$e_{j}=-k\sum_{i=1}^{n}P_{ij}\ln\left(P_{ij}\right)$$ (8)

$$g_{j}=\frac{1-e_{j}}{m-E_{e}}$$ (9)

$$W_{j}=\frac{g_{j}}{\sum_{j=1}^{m}g_{j}}$$ (10)

where *X*_*ij*_ is the value of the *j-th* index of the *i-th* oil well, (i = 1,2…,n,j = 1,2,…,m); $X_{ij}^{'}$ is the normalized data for positive indicators; $X_{ij}^{''}$ is the normalized data of the negative index, $X_{ij}^{'}$ and $X_{ij}^{''}$ are still denoted as *X*_*ij*_; *P*_*ij*_ is the proportion of the *i-th* sample value under the *j-th* index; *e*_*j*_ is the moisture value of the *j-th* index, $E_{e}=\sum_{j=1}^{m}e_{j}$; *g*_*j*_ is the coefficient of variance for the *j-th* index, 0≤*g*_*j*_≤1, $\sum_{j=1}^{m}g_{j}=1$; and *W*_*j*_ is the weight of the *j-th* index

#### Selection of the initial cluster centre

For the iterative clustering k-means algorithm, when the initial cluster centre and the final cluster centre differ significantly, the number of iterations of the algorithm will increase. Therefore, it is very important to select a suitable initial cluster centre. The k-means algorithm randomly selects the initial clustering centres; however, most of the clustering centres of the dataset are distributed in the higher data density range. If the randomly selected initial cluster centres are distributed at the boundary, inaccurate results may be produced. Therefore, when using the density-clustering algorithm to select a suitable initial clustering centre for the improved clustering algorithm, k objects with higher density will be selected to replace the randomly selected initial clustering centre. The specific steps to accomplish this are as follows:

Step 1: For any point in a given dataset, calculate the weighted Euclidean distance to the remaining points and sort them in ascending order to obtain the distance set M. Set MinPts to k, and use the k-th distance of the distance set M as the k-distance of the point. Calculate the k-distance of all points to form the k-distance set, and draw an image of the k-distance set to find the point with the most intense change, that is, the required neighbourhood radius ε.

Step 2: In all data samples, if there are no less than Minpts objects in the ε-neighbourhood of a point, that point is the core object, and the core object set X_i_ is generated in this manner.

Step 3: Find all the points with reachable density points from any core point in the core object set to generate clusters. This process is repeated until all the core points are visited.

Step 4: Find the average value of the i(1≤*i*≤*k*) cluster as the temporary cluster centre ci, and use formula (11) to calculate the weighted Euclidean distance from each sample *x*_*m*_ to *c*_*i*_ in the i-th cluster;

$$dis\left(x_{m},c_{i}\right)=W_{j}\sqrt{{\left(x_{m}-c_{i}\right)}^{2}}$$ (11)

Step 5: The point closest to the temporary cluster centre is the centre point in the cluster, that is, the initial cluster centre point.

Step 6: Repeat steps 4 and 5 until k initial cluster centres are found.

#### Model logic block diagram

**Fig 1:**
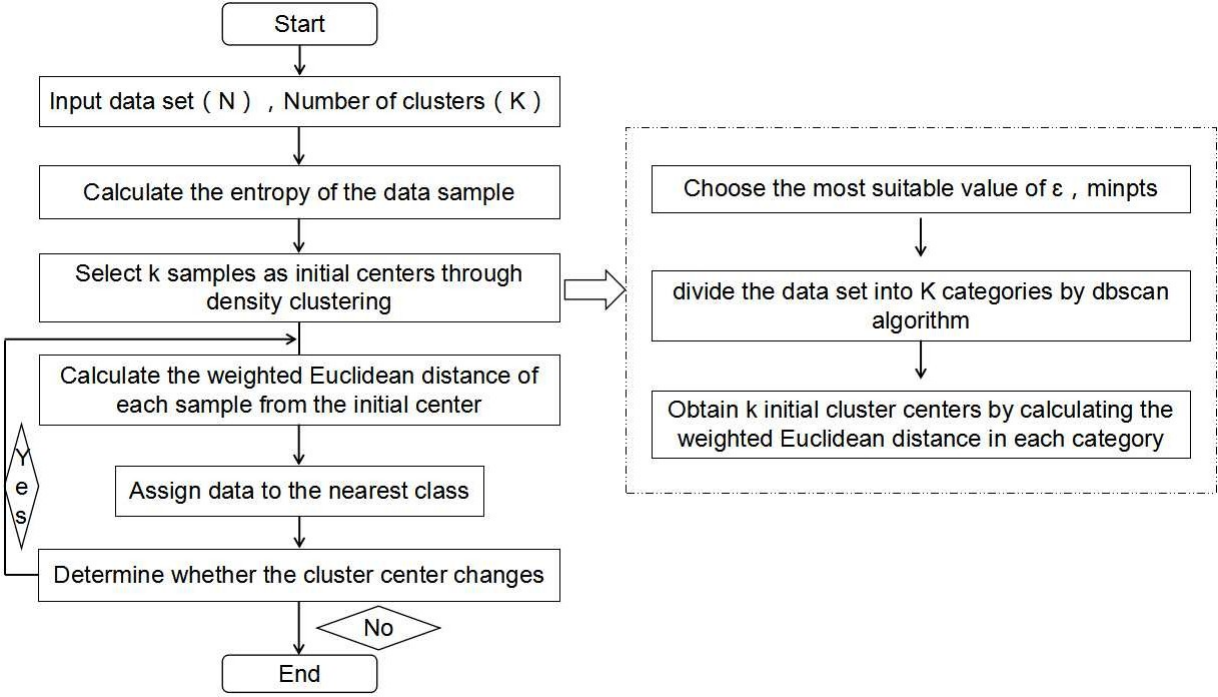
Improved k-means algorithm flow chart.

According to the principle of the improved k-means algorithm, a clustering analysis process was developed–as shown in Fig 1 –and the weighted k-means clustering analysis algorithm program combined with density clustering was compiled according to the aforementioned flowchart to perform cluster analysis on the block oil well parameter observation set.

For cluster analysis, determining the number of clusters is very important. This article comprehensively considers several important factors such as similarities within groups, differences between groups, the number of clusters and sample size to determine a reasonable number of clusters. To reflect the characteristics of the data itself, this paper uses entropy to weight the Euclidean distance, such that each factor can integrate its own value and the weight in the block for distance calculation and cluster analysis. In fact, the cluster centres are usually distributed in the range of the high density of data objects; therefore, this study uses density clustering to determine the initial cluster centres. By combining density clustering and the weighted k-means algorithm, it is expected that the dataset can be better clustered into several categories based on its own data characteristics.

## Analysis of the results

### Determining the number of clusters

**Fig 2:**
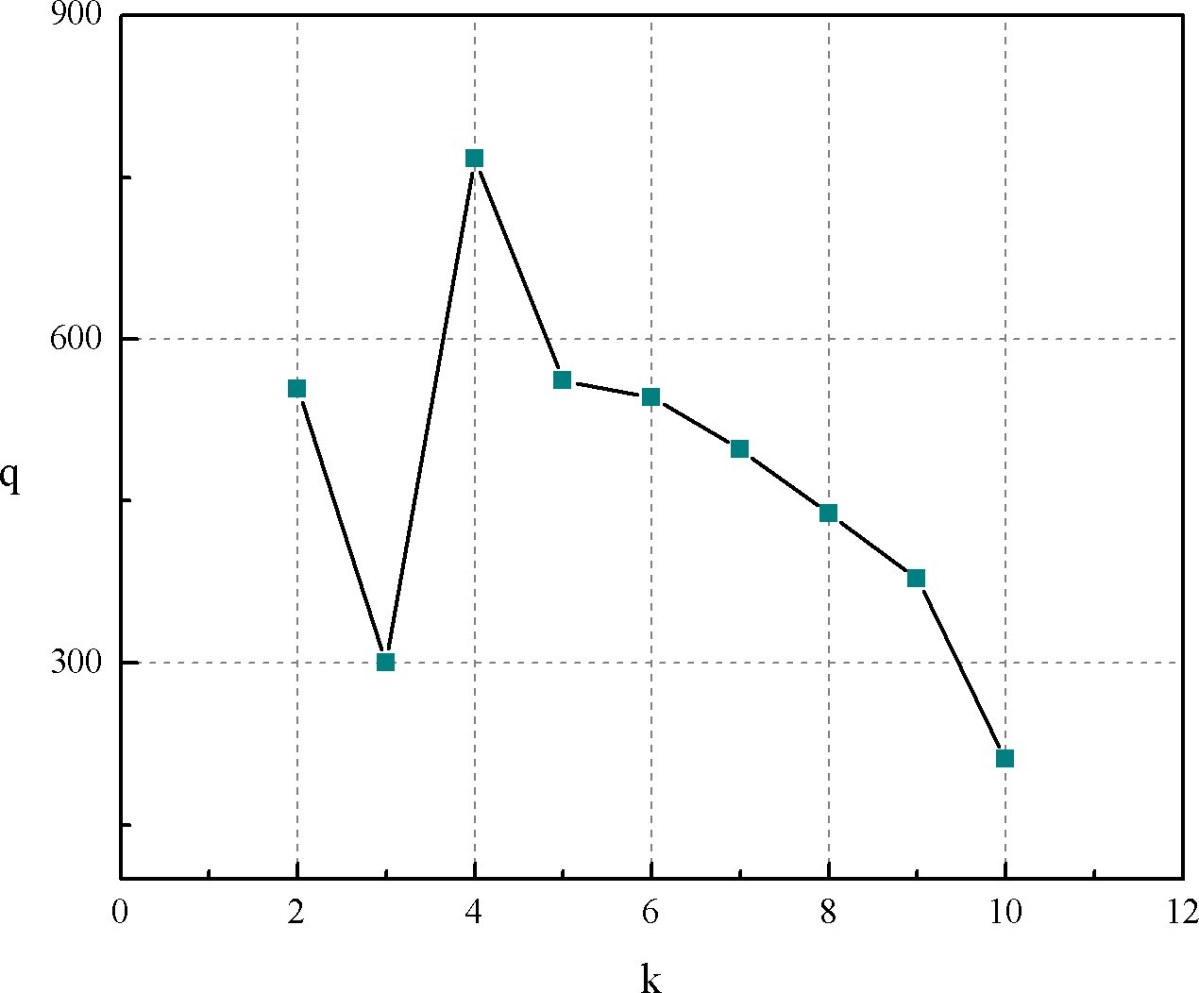
Selection of the best k value.

The ideal number of clusters for the block oil field is 3–5, based on the clustering of low-efficiency wells, normal wells and high-efficiency wells. If the number of clusters is too small, they do not sufficiently reflect the characteristics of the block data. If the number of clusters is too large, the characteristics are too detailed, making interpretation cumbersome. According to formula (4), the k value should range between 1 and 10, facilitating the drawing of a graph, as shown in Fig 2. It is clear from the formula that the larger the ratio, the better. From the figure, it can be seen that when the k value is 4, the coefficient curve has the highest peak point, that is, it is determined that the cluster number coefficient that has the maximum value. The number of classes is four.

**Fig 3:**
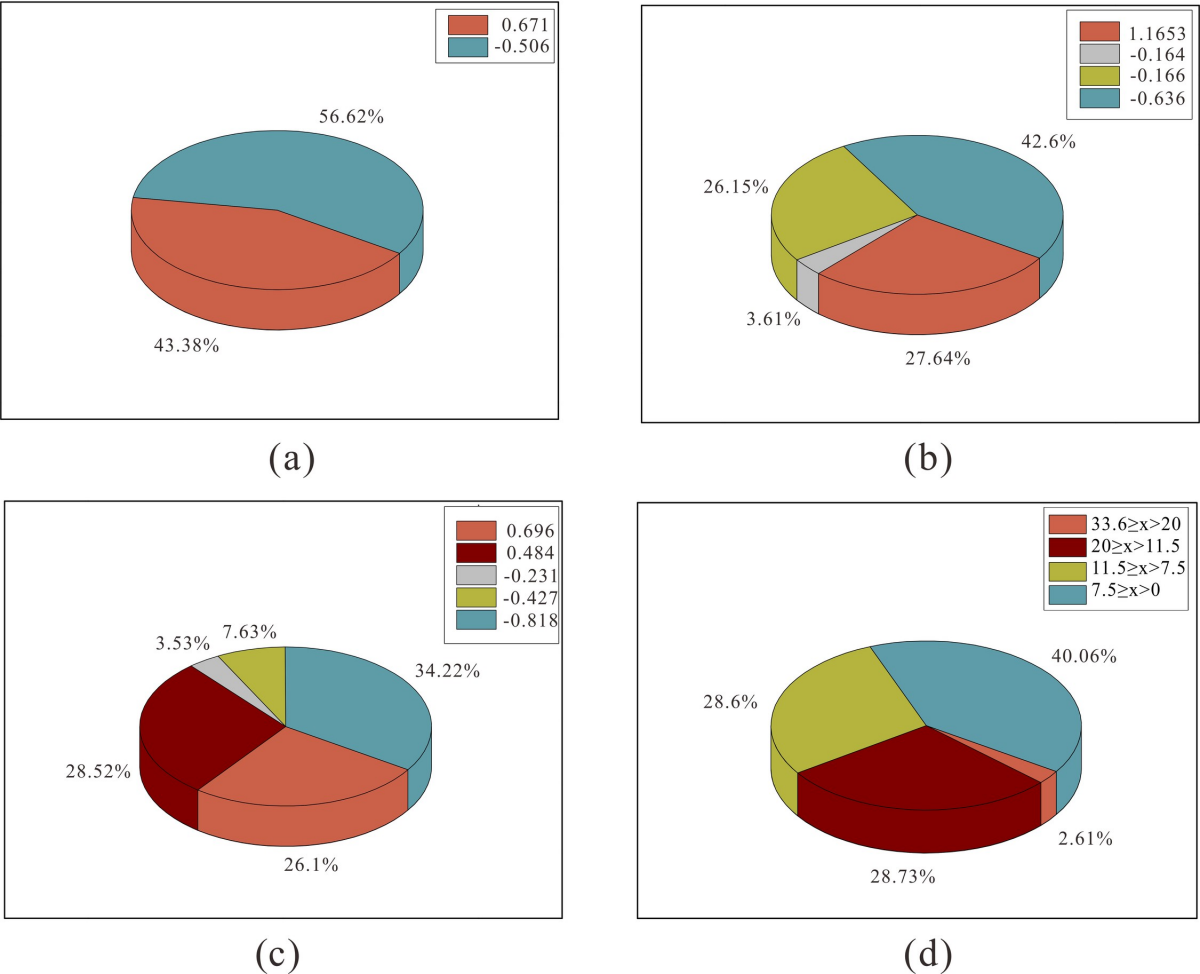
Comparison of system efficiency ratios (a) k = 2, (b) k = 3, (c) k = 4 and (d) raw data.

To determine the optimal number of clusters, the clustering results with k values of 2, 4 and 5 were analysed and compared. The data of each well and each month is treated as a single data row and the percentage of system efficiency of different cluster numbers is compared with the original data. This is shown in the pie chart in Fig 3. The average value of the system efficiency in this block is 9.5%, and the average value of the system efficiency after the clustering calculation is 0 after dimensionless processing. As can be seen from the figure, in the original data, the system efficiency is between 7.5% and 11.5% in normal wells, which account for 28.6% of all wells; those with efficiencies above 11.5% are collectively referred to as high-efficiency wells, which account for 31.34% of the total; and those with efficiencies below 7.5% are collectively referred to as inefficient wells, which account for 40.06% of the total. When the k value is 2, the wells are only divided into high-efficiency and low-efficiency groups, accounting for 43.38% and 56.62% of the total, respectively, which does not meet the assumption for cluster analysis in this block. When the k value is 5, the two groups for the system efficiency dimensionless quantities of -0.231 can be collectively called normal wells, which account for 3.53% of the total; and the two groups with efficiencies of 0.696 and 0.484 can be collectively referred to as high-efficiency wells, which account for 54.62% of the total, the proportion of inefficient wells is 41.85%, in terms of proportion, it cannot be the number of block data clusters. When the k value is 4, the two groups for the system efficiency dimensionless quantities of -0.164 and -0.166 can be collectively called normal wells. Normal wells account for 29.97% of the total, low-efficiency wells account for 42.6% and high-efficiency wells account for 27.64%. The proportion of high-efficiency wells is close to that in the original data. Considering the proportions and determining the cluster number coefficient q, the optimal number of clusters in the block is determined to be 4.

### Weight calculation

**Fig 4:**
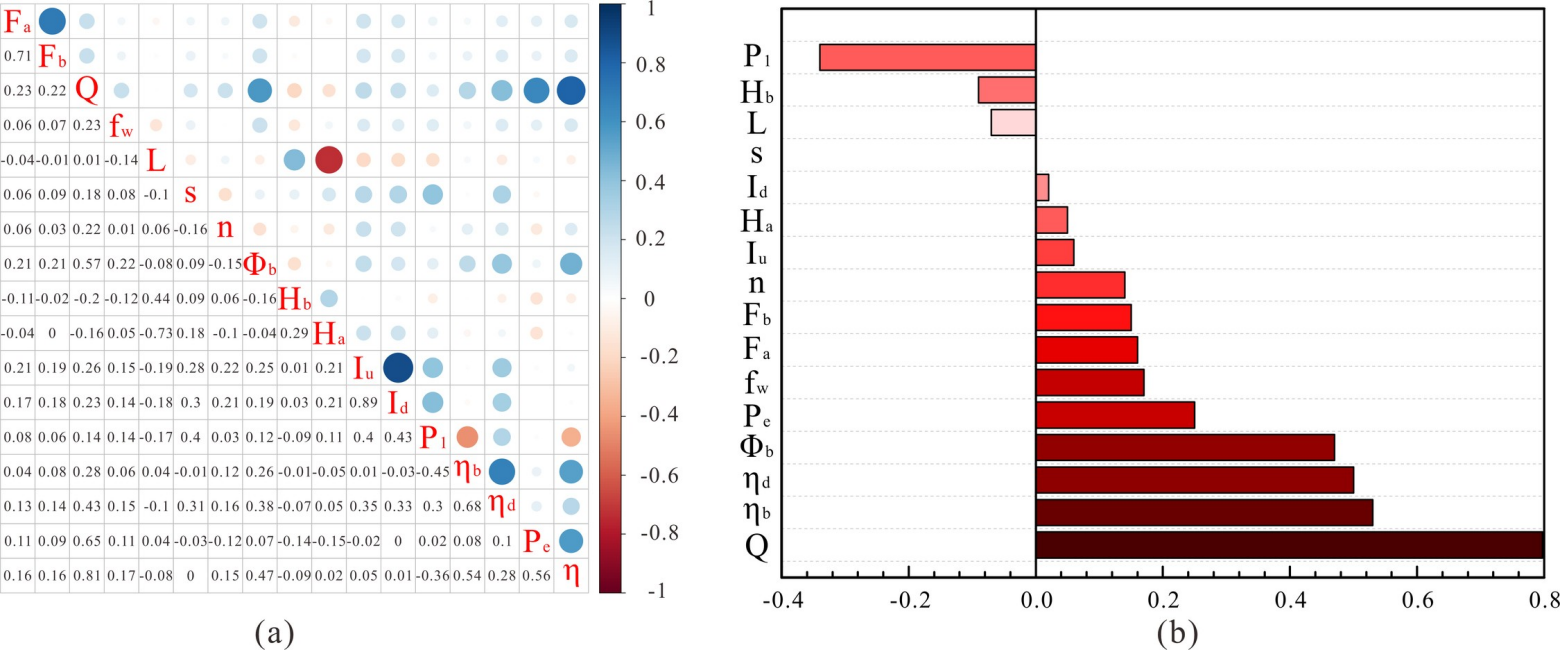
Correlation of feature parameters: (a) correlation between factors; (b) Correlation between various factors and system efficiency.

First, the correlation coefficients among the factors are listed, as shown in Fig 4(A). It can be seen that there is a strong correlation between certain factors, which proves that the system efficiency is not only related to direct factors, but also affected by some indirect factors. For example, the system efficiency has a strong correlation with the daily liquid production, and the daily liquid production is also correlated with the pump diameter, and motor power consumption; therefore, the latter two factors also indirectly affect the system efficiency. For this reason, it is necessary to calculate the entropy value of each factor and evaluate its importance in terms of the entire system. Next, the correlation between each factor and system efficiency is extracted, as shown in Fig 4(B)–it can be seen that the input power, pump depth and submersion degree have an inverse relationship with system efficiency. Based on this, the block data is standardized prior to entropy calculation.

**Fig 5:**
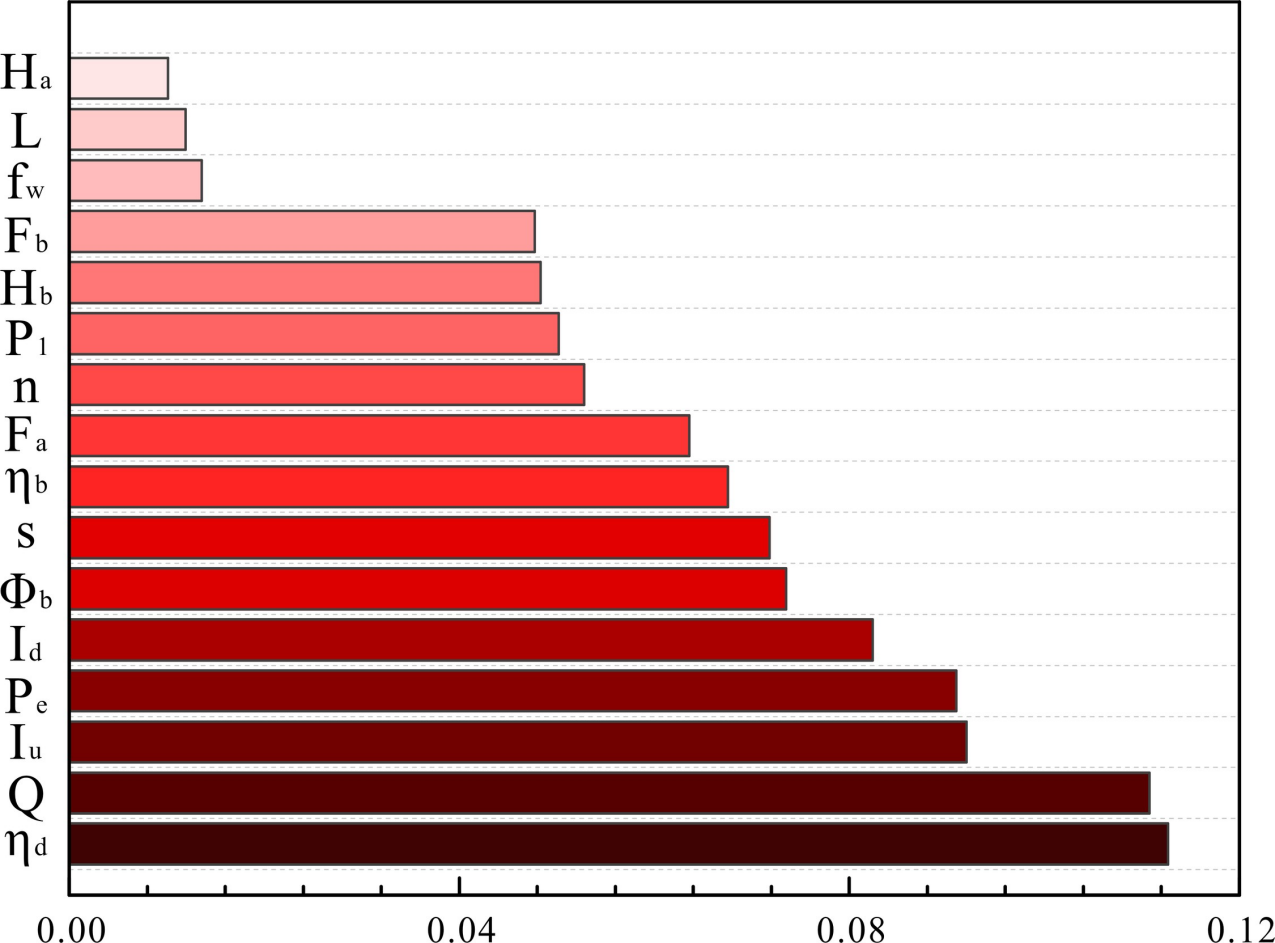
Histogram of the weight of each factor.

The smaller the entropy value, the larger the information utility value. To better represent the proportion of each factor in the overall oil well data of the block, the difference coefficient of the j-th index is calculated using formula (9). The greater the difference in the index value, the greater the impact on the program evaluation. The ratio of the difference coefficient of each factor and the overall difference coefficient is defined as the weight of the factor; the weights of each factor are shown in Fig 5. It can be seen that the motor utilization and daily fluid production have high weights, while the water content and liquid depth and submergence have relatively small weights. The weight of each factor is used as the coefficient for calculating the Euclidean distance.

### Determining the initial cluster centre

**Fig 6:**
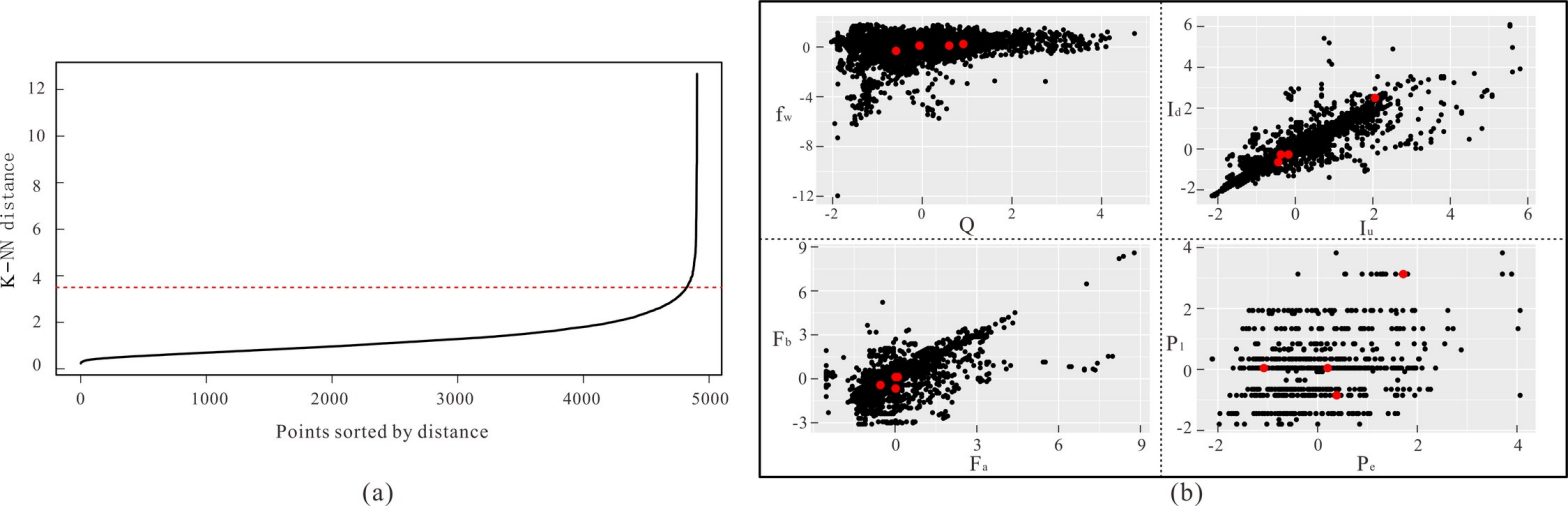
Initial cluster centre determination: (a) ε value determination; (b) Initial cluster centre plot.

The parameter ε and minPts in the dbscan algorithm must be set first, that is, the minimum number of observation points included in the neighbourhood radius and the radius of the field, respectively. Setting a small radius would not allow the data to cluster properly, and setting a radius too large would cause significantly differing data to cluster; therefore, the proper selection of the radius is very important. On the basis of the number of clusters being 4, the k-distance curve is drawn as shown in Fig 6(A). The obvious inflection point in the k-distance curve is a better radius parameter, which can be inferred from the figure; it is most appropriate when the ε value is 3.5. The initial clustering centre selected by the dbscan algorithm is shown in Fig 6(B). It can be seen that the initial clustering centre is in the higher density area in the four scatter plots.

### Comparative analysis of the improved algorithm and k-means algorithm results

**Fig 7:**
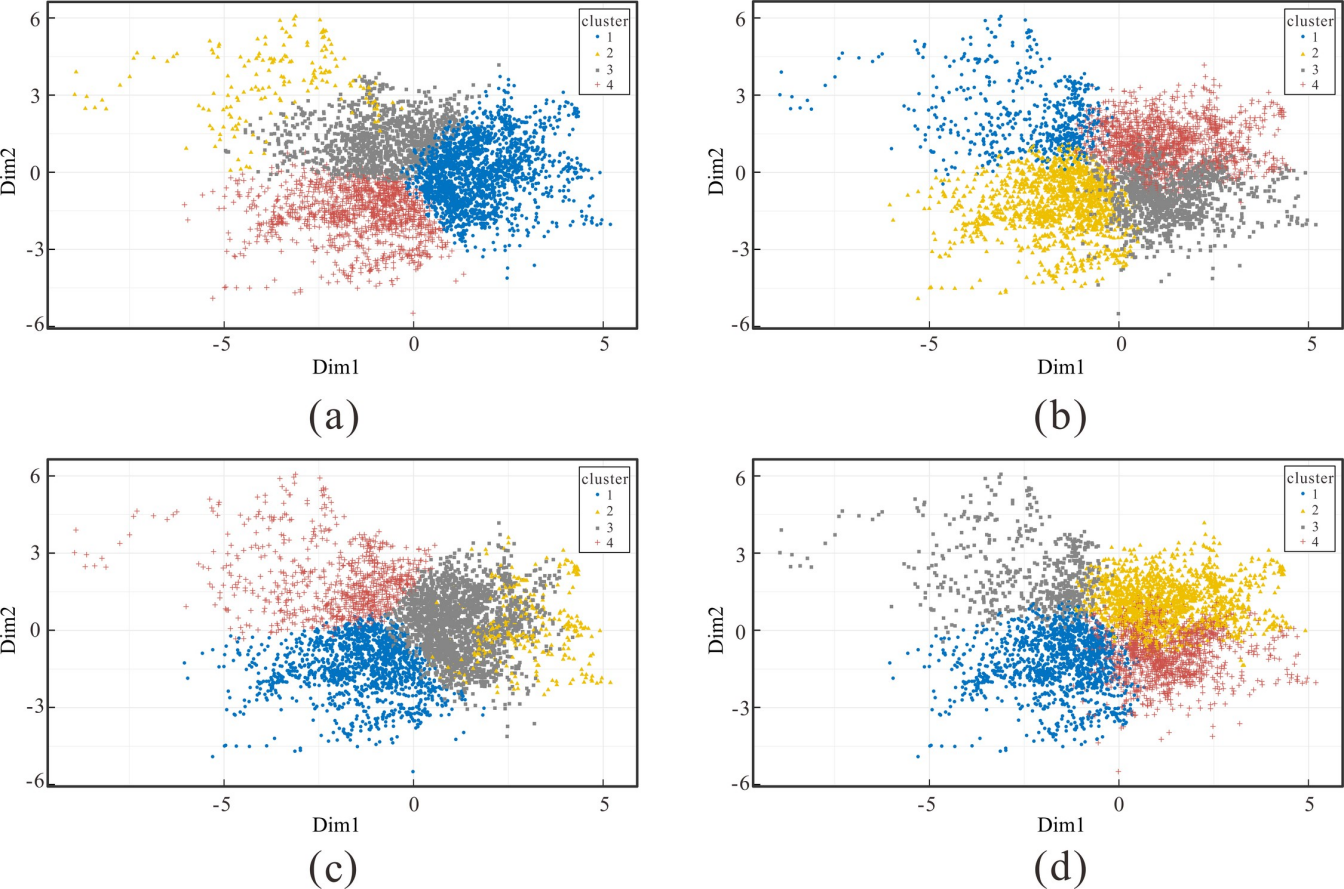
Comparison of the results of the improved algorithm and k-means algorithm: (a) improved algorithm; (b) k-means algorithm (first test); (c) k-means algorithm (second test); (d) k-means algorithm (third test).

Since the initial clustering centre of the k-means algorithm is randomly selected, the results of k-means clustering are different in each iteration. The three test results of the improved clustering algorithm and the original k-means algorithm are compared and analysed, as shown in Fig 7. It can be seen that the clustering results of the improved algorithm are better than those of the k-means algorithm. The results of the improved algorithm overlap less and the boundaries of the categories are more distinct. It can be seen from Fig 7(A) that the overall conformity with the expected assumptions only has a small overlap. The system efficiency dimensionless values of the four groups are 1.165, -0.164, -0.166 and -0.636. The second and third groups can be collectively referred to as normal wells. The proportions of high-efficiency, normal and low-efficiency wells are 27.64%, 29.76% and 42.6%, respectively–this does not differ much from the original data. As can be seen in Fig 7(B) and Fig 7(D), the clustering effect is not ideal, and there is significant overlap between different groups. It can be seen from Fig 7(C) that the clustering effect is improved. The dimensionless values of the system efficiency in the four groups are 1.11, -0.123, -0.584 and -0.936, and their proportions are 30.45%, 18.12%, 47.04% and 4.39%, respectively, which differ from the original data.

Table 3 shows the comparison of the sum of squared errors between the improved algorithm and kmeans algorithm. Both algorithms aggregate the oil field block data into four groups. It can be seen that the three results of kmeans algorithm are different, which prove the instability of kmeans algorithm results, but the sum of squared errors between groups are larger than the value of the improved algorithm, which shows that the clustering results of the improved algorithm are more similar between groups.

**Table 3:** Comparison of within-group error sum of squares.

Groups	1	2	3	4
Improve algorithm	22994.58	3604.16	8101.387	12888.65
Kmeans-1	16035.71	10121.28	15769.30	16001.83
Kmeans-2	17537.47	10105.44	14284.18	15630.76
Kmeans-3	19124.50	5069.61	15839.66	17708.55

Table 4 shows the comparison between the center distances of different groups in the results of the improved algorithm and kmeans algorithm. It can be seen that the distance between the groups of the improved algorithm is larger than that of the kmeans algorithm, indicating that the difference between the groups of the improved algorithm is stronger. In general, each effect of the original k-means algorithm is random; some effects can meet expectations, but there is no guarantee that each effect performs well. The improved algorithm better combines the characteristics of the block oil field data itself, because the positive and negative influence and weight of the factors are considered. Selecting initial cluster centers through density clustering can solve the initial center sensitivity problem of k-means algorithm. The improved algorithm can highlight the differences between different categories and provide more accurate results.

**Table 4:** Distance between groups.

Algorithm	Groups	2	3	4
Improve algorithm	1	6.84	3.08	3.06
	2	-	5.51	5.81
	3	-	-	2.60
kmeans	1	3.55	3.08	3.01
	2	-	3.84	5.04
	3	-	-	2.44

**Fig 8:**
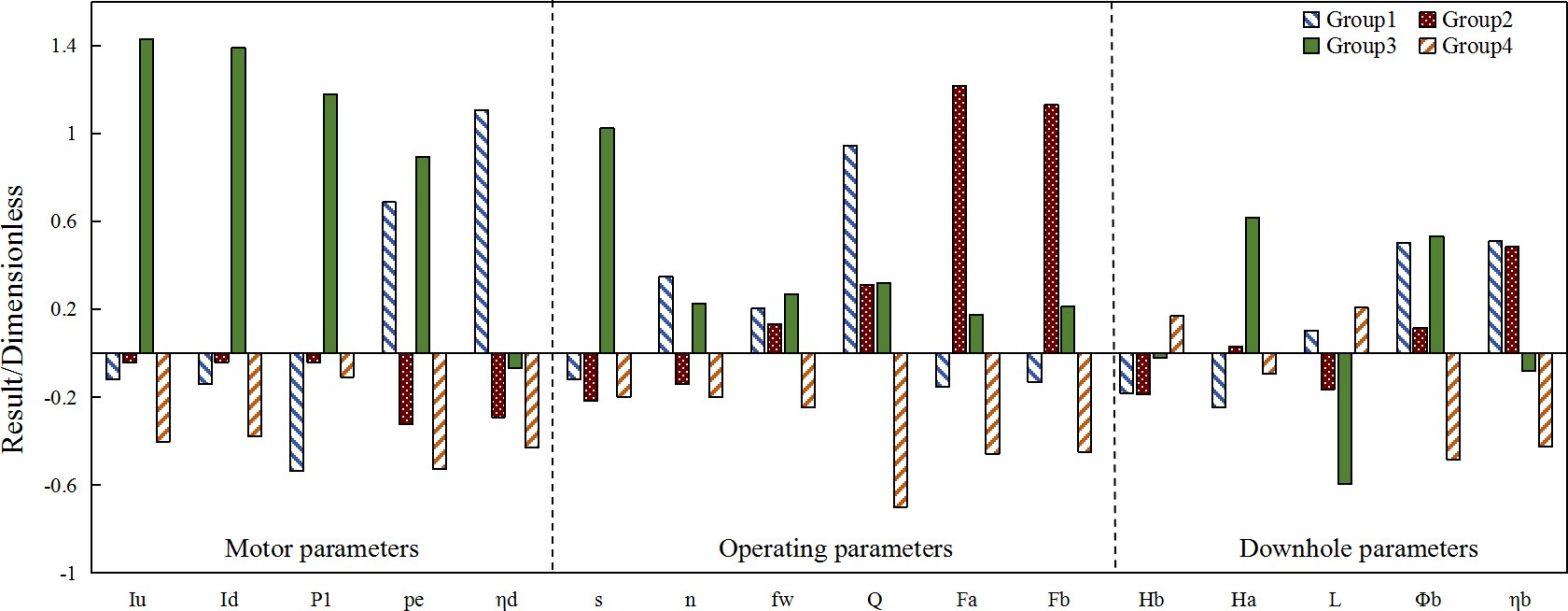
k-means clustering results.

Fig 8 shows the analysis results for the clustering number of 4 in block oil well data using the k-means algorithm. Group 2 (η = -0.206) and group 3 (η = -0.133) can be collectively referred to as normal wells. It can be seen from the figure that the input power and liquid depth of group 1 (η = 1.209, high-efficiency wells) have the lowest value, and the motor utilization, daily fluid production, frequency, and pump efficiency have the highest values. In group 4 (η = -0.654, low-efficiency wells), the maximum current of the upstroke, maximum current of the downstroke, power consumption, motor utilization, frequency, water content, daily fluid production, oil pressure, casing pressure, pump diameter and pump efficiency have the lowest values, of which only the pump diameter, water content and daily liquid productionare lower than the average values for the block data. Through a comprehensive analysis, it can be seen that, in the k-means clustering results, the motor utilization, daily fluid production and pump efficiency are significantly higher than those of other wells. For low-efficiency wells, water content, pump diameter, daily fluid production are lower than the average values of the block. Observation and analysis show that pump efficiency, daily fluid production and system efficiency have an obvious positive correlation, therefore, these factors should be improved in increase system efficiency.

**Fig 9:**
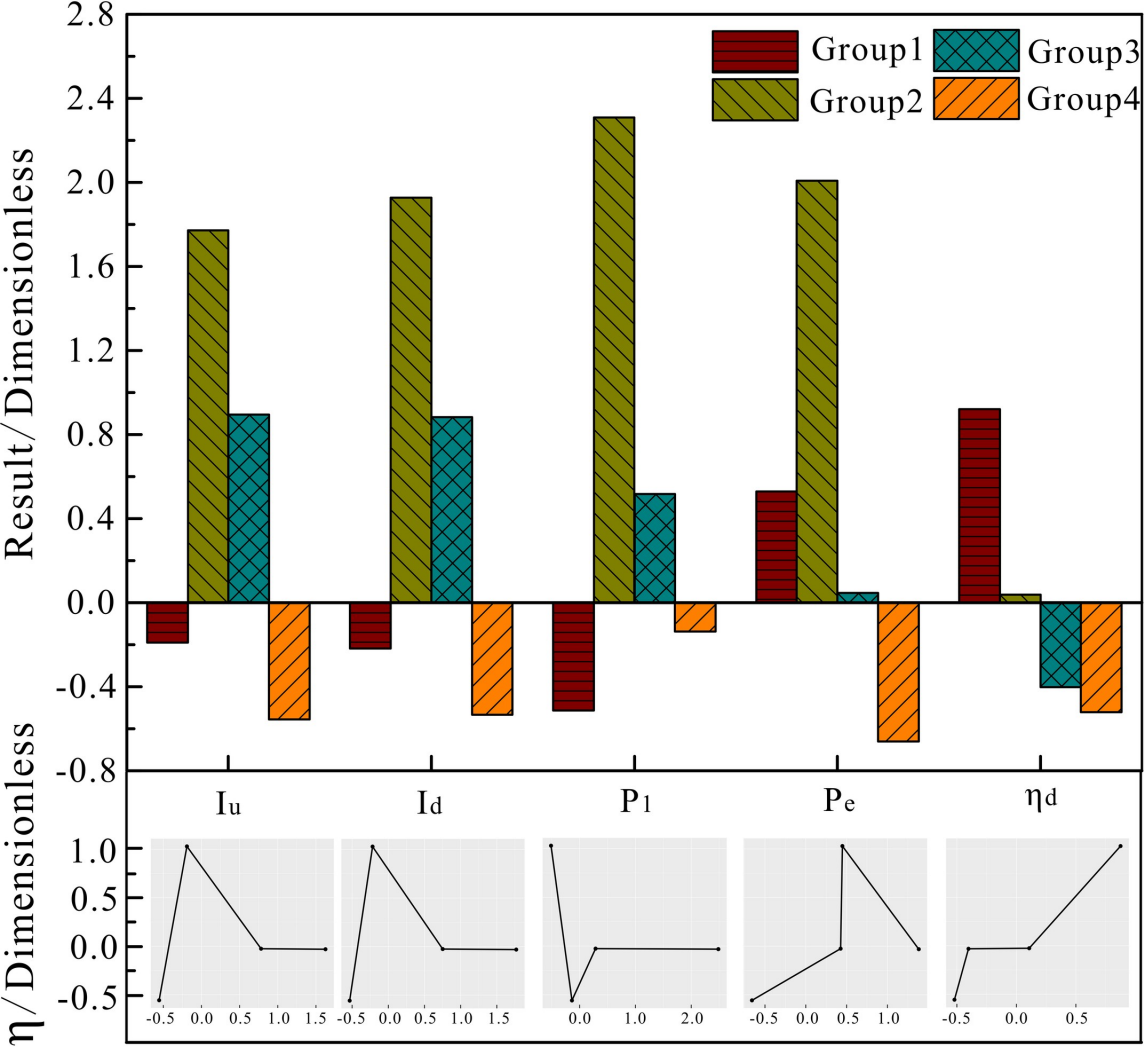
Block motor parameters for the improved algorithm clustering analysis.

**Fig 10:**
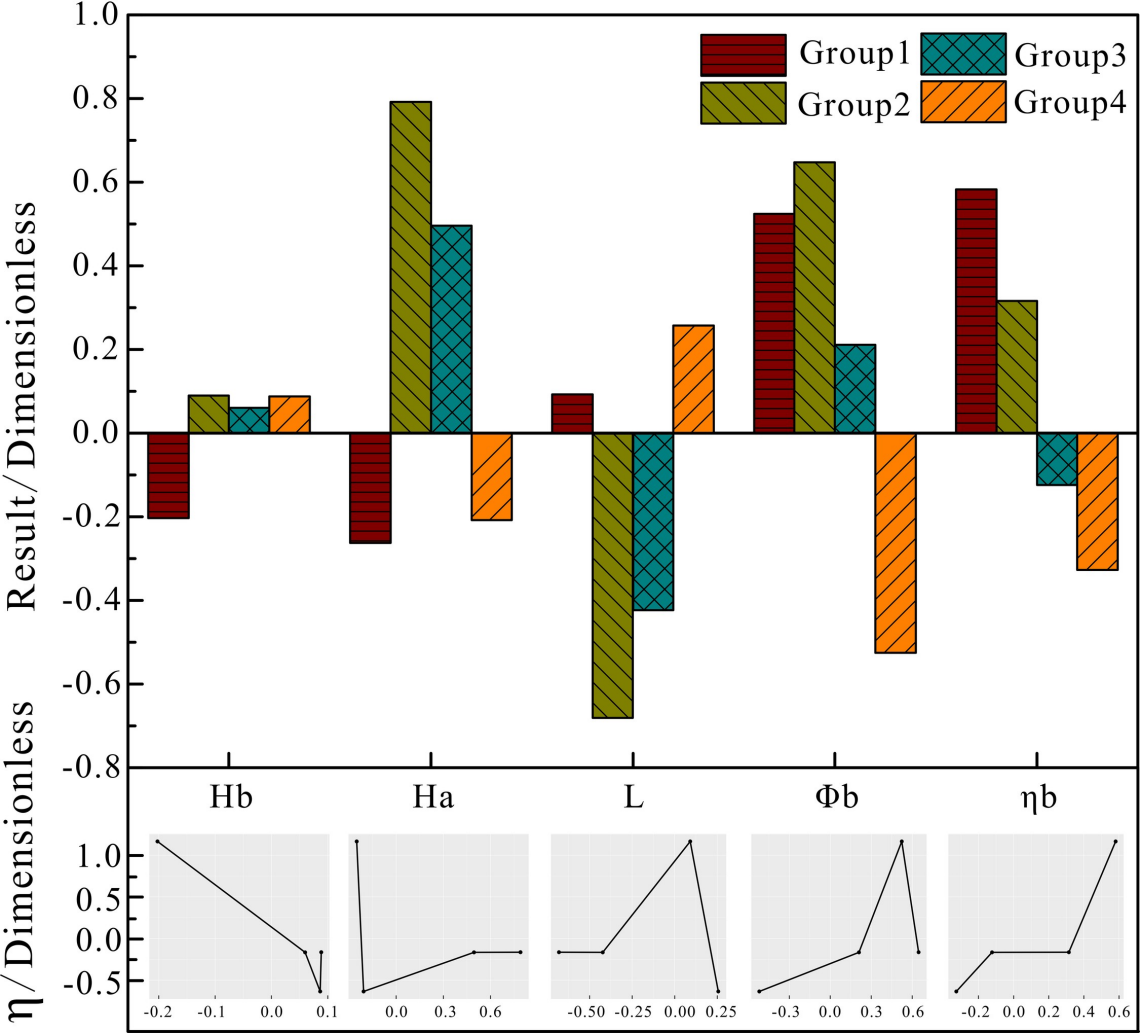
Block downhole parameters of the improved algorithm cluster analysis.

**Fig 11:**
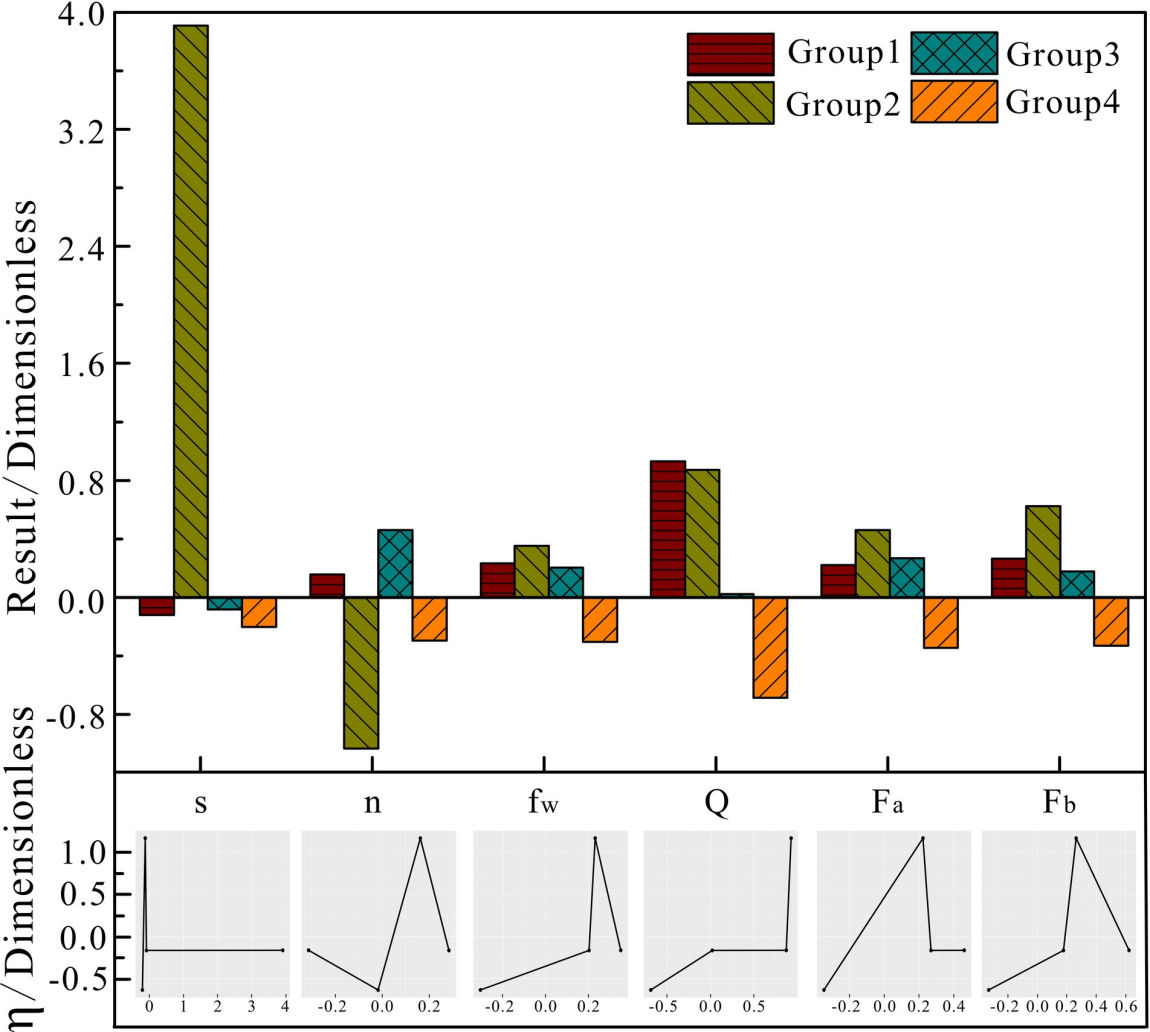
Block operating parameters of the improved algorithm clustering analysis.

Figs 9 to 11 show the analysis results when using the weighted k-means algorithm combined with density clustering on block oil well data, with a cluster number of 4. The upper part is a histogram of each parameter produced by the improved algorithm, and the lower part is a graph corresponding to the system efficiency according to various factors. It can be seen from the clustering results that the group 1, with a dimensionless value of 1.165, includes high-efficiency wells, accounting for 27.64% of the total; the efficiencies of group 2 and group 3 are -0.164 and -0.166, respectively, which are referred to as normal wells, accounting for 3.61% and 26.15% of the total, respectively; and group 4, with an efficiency value of -0.636, include low-efficiency wells, accounting for 42.6% of the total.

Fig 9 shows the clustering results of the improved algorithm for the observation set of oil well parameters in the block. It can be seen from the figure that the maximum current and input power of the upper and lower strokes of group 1 are lower than the average values; the input power is the lowest value of the four groups, and the output power and motor utilization rate are higher than the average values, where the motor utilization rate is the highest value of the four groups. All motor parameter values in group 4 are lower than the overall average values, and the maximum current of the upstroke, maximum current of the downstroke, power consumption and motor utilization rate are the lowest among the four groups, of which only the power consumption of group 4 is lower than the average value for the block. Combined with the analysis of the graph, it can be seen that there is a positive correlation between motor utilization and system efficiency, that is, the higher the motor utilization, the higher the system efficiency. The trends of change of the maximum current of the upstroke and the maximum current of the downstroke are almost the same, both of which increase the system efficiency as they increase, subsequently making it decrease and plateau. The curve of input power and system efficiency first declines, then rises and finally flattens, that is, there is a suitable interval for the maximum current and input power of the upper and lower strokes where the block has a higher system efficiency.

The downhole parameters in the clustering results of the improved algorithm for the observation set of oil well parameters in the block are shown in Fig 10. The pump depth and liquid depth of group 1 are lower than the average values, and are the lowest among the four groups; the dimensionless value of pump depth of group 1 is lower than the block average. However, the submergence, pump diameter and pump efficiency are significantly higher than the average values–the pump efficiency is the largest among the four groups. The dynamic liquid level, pump diameter and pump efficiency of group 4 are lower than the average values for the block. Among them, the pump diameter and pump efficiency are the lowest of the 4 groups, and the pump diameter values of the four groups are only lower than the average value of group 4. From these results and those of the graph, it can be seen that the pump efficiency is positively correlated with the system efficiency, that is, increasing the pump efficiency can increase the system efficiency. The graphs of dynamic liquid level, pump depth and system efficiency show a trend of declining first and then increasing, that is, the two parameters have an interval where the system efficiency is lower than the average value, the specific values of which need to be further investigated. It can be determined that the downhole characteristics of the high-efficiency wells are that the dimensionless value of pump efficiency is the highest value among the four groups, and the value of pump depth is lower than the average value; the downhole parameters of low-efficiency wells are characterized by the pump diameter being lower than the average value, and the pump diameter and pump efficiency being the lowest among the four groups. To improve the efficiency of the pumping unit system in this block, the pump efficiency and pump diameter should be increased, while the pump depth should be decreased.

The operating parameters of the improved algorithm’s clustering results on the observation set of oil well parameters in the block are shown in Fig 11. In group 1, the frequency, water content, daily fluid production, oil pressure and casing pressure are all higher than the block average. All operating parameter values of group 4 are lower than the overall average, and among the four groups, only group 4 has water content, daily fluid production, oil pressure and casing pressure lower than the overall average. It can be seen from the graph that the daily fluid production volume, is positively correlated with the system efficiency, that is, the system efficiency increases with increases in the daily fluid production volume; the stroke, oil pressure, casing pressure and system efficiency graphs all increase first and subsequently decline, indicating that these three factors have a certain region in which the system efficiency is higher than the average value. The specific value for this needs to be further investigated. From the clustering results, it can be seen that the low values of the water content, daily fluid production, oil pressure and casing pressure are the reasons for the reduction in the efficiency of the pumping unit system.

A comprehensive analysis of Figs 8 to 11 compares the clustering results of the improved algorithm with those of the k-means algorithm. It can be seen that the ordinate interval of the improved algorithm is larger than that of the k-means algorithm. The greater the distance, the more obvious the clustering result of the improved algorithm. The two algorithms have certain things in common, and both show that the motor utilization rate, pump efficiency, daily fluid output and system efficiency have an obviously positive correlation. In the k-means clustering results, the main characteristic of low-efficiency wells is that the motor parameters and operating parameters are all lower than the average value. Among the four groups, only the low-efficiency well groups have lower water content and daily fluid production than the block average; the most important feature of high-efficiency wells is that the input power is significantly lower than that of other well groups, and the motor utilization, daily fluid production and pump efficiency are significantly higher than those of other wells. In the improved algorithm results, the significant feature of inefficient wells is that the motor parameters and operating parameters are lower than the average values. Among the four groups, only the dimensionless values of water content, daily fluid production, oil pressure, casing pressure, power consumption and pump diameter of the inefficient well groups are lower than those of the block average; the obvious characteristics of high-efficiency wells are that the motor utilization rate, pump efficiency and daily fluid production are the four highest values; the input power has the four lowest values; and the dimensionless value of the pump depth is lower than the block average. Most of the clustering results of the two algorithms are the same. In contrast, the improved algorithm has more obvious characteristics for high-efficiency wells and low-efficiency wells, which shows that the improved algorithm can better reflect the characteristics of oil wells in the block.

## Conclusion

1. A weighted k-means algorithm combined with density clustering was proposed in this study. First, an appropriate number of clusters is selected using the formulas for the sum of squares of errors between groups, sum of squares of errors within groups, number of clusters and sample size. Next, density clustering is used to select the initial cluster centre. Finally, the weight of each factor is obtained by calculating the entropy value, which is used as the coefficient of the improved clustering algorithm to calculate the Euclidean distance.
2. The improved clustering algorithm and the original k-means algorithm are used to perform cluster analysis on the motor parameters, downhole parameters and operating parameters of the block oilfield. The results of the two algorithms are compared; the k-means algorithm cannot guarantee the accuracy of each clustering result, and the improved algorithm has obvious classification boundaries and stable clustering results. The analysis results show that the improved algorithm is more suitable for the cluster analysis of block oilfield data.
3. The similarities between the k-means and improved algorithm clustering results include the pump efficiency, daily fluid production and system efficiency having an obviously positive correlation. The main characteristics of low-efficiency wells are, pump diameter, water content and daily fluid production being lower than the block average. In comparison to the clustering results of the k-means algorithm, the improved algorithm has more features: the motor utilization and system efficiency having an obviously positive correlation, the pump depth of high-efficiency wells is lower than the block average, and the daily fluid production is higher than the block average. Further, the oil pressure, casing pressure and power consumption, of low-efficiency wells are lower than the block average.
4. Using the improved algorithm clustering results, the measures to improve the efficiency of the block system are summarized. In terms of motor parameters, the power consumption should be increased to increase the input power, which, in turn, increases the utilization rate of the motor. In terms of operating parameters, relevant operations should be carried out to indirectly increase the water content, daily fluid production, oil pressure and casing pressure. In terms of downhole parameters, pump efficiency should be improved and the depth of the pump should be reduced under the condition of satisfying submergence.

## Supporting information

S1 File (XLSX)
